# Synergistic Effects of Thyroxine and Nano‐Melatonin on Post‐Thaw Sperm Quality, Antioxidant Defence and Fertility of Cryopreserved Simmental Bull Semen

**DOI:** 10.1002/vms3.71252

**Published:** 2026-09-30

**Authors:** Marwa Abonagy, Ahmed Arisha, Maha Salama, Mohey Ashour, Mustafa Shukry, Sameh Shedeed, Ahmed Shehabeldin, Nagi El‐Shafai, Zein Ibrahim, Shawky Mahmoud, Michel Saad, Ahmed Alameen, Rasha Yassin Elkhidr, Osama Hassan Alif, Arafat Khalphallah, Enas Elmeligy, Khaled Abdulaziz Khesruf, Enas Ahmed Abdelhafez, Alaa Algabary

**Affiliations:** ^1^ Department of Animal Physiology and Biochemistry, Faculty of Veterinary Medicine Badr University in Cairo (BUC) Badr City Cairo Egypt; ^2^ Department of Physiology, Faculty of Veterinary Medicine Zagazig University Zagazig Egypt; ^3^ Animal Reproduction Research Institute (ARRI) Agricultural Research Center (ARC) Giza Egypt; ^4^ Department of Biotechnology Research, Animal Production Research Institute (APRI) Agricultural Research Center (ARC) Dokki Egypt; ^5^ Riwina Animal Production Farm, Agricultural Research Center (ARC) Ministry of Agriculture Egypt; ^6^ Department of Biomedical Sciences, College of Veterinary Medicine King Faisal University Al‐Ahsa Saudi Arabia; ^7^ Nanotechnology Center, Chemistry Department, Faculty of Science Kafrelsheikh University Egypt; ^8^ Department of Physiology Faculty of Veterinary Medicine Kafrelsheikh University Kafrelsheikh Egypt; ^9^ Department of Clinical Sciences College of Veterinary Medicine King Faisal University Al‐Hofuf Saudi Arabia; ^10^ Department of Physiology Faculty of Veterinary Medicine University of Khartoum Shambat Sudan; ^11^ Division of Internal Medicine, Department of Animal Medicine Faculty of Veterinary Medicine Assiut University Assiut Egypt; ^12^ Veterinary Teaching Hospital, Faculty of Veterinary Medicine Assiut University Assiut Egypt; ^13^ Department of Animal diseases Faculty of Veterinary Medicine Hama University Hama Syria; ^14^ Department of cell and tissues Faculty of Veterinary Medicine Assiut University Assiut Egypt

**Keywords:** cryopreservation, fertility, nano‐melatonin, simmental bull, thyroxine

## Abstract

**Background:**

Cryopreservation induces oxidative stress and functional damage in spermatozoa, negatively affecting sperm quality and fertility. Nano‐melatonin (NM) and thyroxine (T) have been suggested as potential cryoprotective agents because of their antioxidant and metabolic regulatory properties.

**Objectives:**

This study aimed to evaluate the effects of thyroxine and nano‐melatonin supplementation in semen extenders on post‐thaw sperm quality, antioxidant status, apoptosis, and fertility of cryopreserved Simmental bull semen.

**Methods:**

Ninety‐six ejaculates were collected from six healthy Simmental bulls over 8 weeks. Semen samples were pooled and diluted with extenders supplemented with T (0.5, 0.75 and 1 µg/dL), NM (30, 45 and 60 µg/dL), or their combinations. Post‐thaw sperm motility, kinematics, viability, acrosome integrity, plasma membrane integrity, mitochondrial membrane potential, DNA integrity, antioxidant parameters, lipid peroxidation, apoptosis‐like changes, and conception rates were evaluated.

**Results:**

The combined thyroxine and nano‐melatonin treatment (T0.5 + NM30) significantly improved (p < 0.05) sperm motility and velocity parameters (VAP, VCL and VSL) compared with the other groups. This group also exhibited significantly higher viability, acrosome integrity, plasma membrane integrity, mitochondrial membrane potential, and DNA integrity. Antioxidant activity (GPx, SOD and TAC) was significantly enhanced, whereas MDA levels and late apoptotic sperm cells were reduced in the nano‐melatonin‐treated and combination groups, particularly T0.5 + NM30. Furthermore, conception rates were significantly increased in the NM30 (77.14%) and T0.5 + NM30 (82.86%) groups compared with the control group (48.57%).

**Conclusions:**

Supplementation of semen extenders with thyroxine and nano‐melatonin, particularly the T0.5 + NM30 combination, improved post‐thaw sperm quality, **antioxidant defence and fertility** of cryopreserved Simmental bull semen, indicating a synergistic cryoprotective effect during semen cryopreservation.

## Graphical abstract

Effects of thyroxine (T) and nano‐melatonin (NM) supplementation on cryopreserved Simmental bull semen were evaluated. The combination of thyroxine (0.5 µg/dL) and nano‐melatonin (30 µg/dL) significantly improved post‐thaw sperm motility, membrane integrity, antioxidant status, and fertility while reducing oxidative stress and apoptosis. These findings demonstrate a synergistic cryoprotective effect during semen cryopreservation.

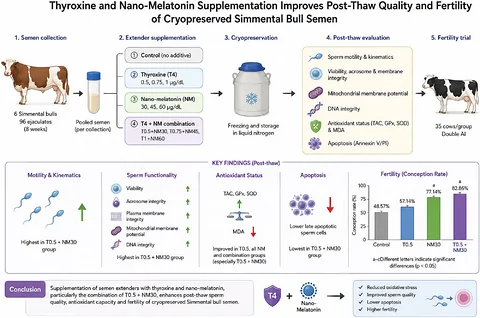

## 1 Introduction

Preserving sperm through freezing for assisted reproduction methods could potentially lead to oxidative stress, damaging the DNA and reducing the chances of successful fertility, mobility and survival of the sperm (Gómez‐Torres et al. 2017). This has sparked a growing interest in finding natural antioxidants that can protect frozen sperm and maintain its overall quality. Melatonin (N‐acetyl‐5‐methoxytryptamine; MT) is an indoleamine hormone synthesised from tryptophan in the pineal gland (Carrasco et al. 2015). MT counteracts the toxic effects of reactive oxygen species by capturing free radicals via G‐protein‐coupled receptors (GPCR). This compound helps maintain cellular function by stimulating antioxidant enzymes and neutralising harmful species, such as hydrogen peroxide, the peroxynitrite anion and nitric oxide (Baş and Nazıroğlu 2019). Recent studies have highlighted melatonin's protective effect on fertility in various animal species (Karimfar et al. 2015; Zhu et al. 2019). Additionally, in humans, melatonin has been shown to be highly beneficial as a sperm cryoprotectant, significantly reducing toxicity and improving recovery of frozen sperm (Ofosu et al. 2021). Evidence suggests that MT improves sperm parameters (plasma membrane integrity, total motility, DNA integrity, and survival rate), while also reducing lipid peroxidation (ChaithraShree et al. 2020).

Nanoparticles have specific features that distinguish them, such as a unique surface area, numerous active surface sites, high surface reactivity, effective catalytic activity, and remarkable adsorption capacity, as stated by Shi et al. (2010). The use of nanoscale particles is becoming increasingly common in the medical field for specific purposes. Developing new drug delivery systems creates opportunities for more precise and efficient treatment, enhancing the therapeutic effects of medications on a molecular level while reducing their adverse effects (Chuffa et al. 2021). Recent research suggests that combining sustained‐release melatonin formulations with nanotechnology can improve effectiveness and safety (Chuffa et al. 2021). Additionally, NM is effective in inhibiting oxidative stress due to its high surface area and small size (Hameed et al. 2024).

The thyroid gland is the body's most vital organ for growth, metabolism and reproduction. It is well recognised that thyroid hormones are crucial for sperm production, steroid hormone synthesis, and testicular growth (Rao et al. 2022). T4 plays a key role in the physiological regulation of energy balance and the maintenance of normal reproductive function in mammals. Thyroxine treatment of semen has been found to enhance the recovery rate of morphologically normal sperm when using both direct and indirect swim‐up approaches (Dhamyaa et al. 2023). It has been demonstrated that T4 rapidly increases the flagellar motions of spermatozoa (hyper motility), leading to an increase in the quantity of spermatozoa recovered by swim‐up (La Vignera and Vita 2018). Recently, Salama et al. (2025) conducted a study in which they added various concentrations of the thyroxine hormone (T4) to the semen of Italian buffalo bulls. They discovered that thyroxine‐treated semen produced the highest viability rate and the lowest abnormalities in frozen‐thawed semen.

Additionally, they observed improved motion and speed measurements, increased conception rates, and cryo‐survival rates in frozen‐thawed cells (Salama et al. 2025). This research aimed to evaluate whether the addition of thyroxine (T) and nano‐melatonin (NM) to bull semen extenders could improve sperm functionality, quality, and antioxidant capacity after thawing, thereby enhancing the bull's in vivo fertility. Although both melatonin and thyroxine have individually demonstrated beneficial effects on sperm function and cryosurvival, their combined use, particularly in nano‐formulated melatonin form, has not yet been investigated in cryopreserved bull semen. Therefore, this study hypothesised that combined thyroxine and nano‐melatonin supplementation may synergistically improve sperm cryotolerance, antioxidant defence, and fertility. The study sought to determine if these supplements at various concentrations would positively influence antioxidant activity, sperm quality, and overall functionality in frozen‐thawed semen. To our knowledge, this is the first study evaluating the combined effects of thyroxine and nano‐melatonin supplementation on cryopreserved bull semen.

## 2 Materials and Methods

The study's experimental design involved collecting ejaculates from six bulls and using a preparation of thyroxine and nano‐melatonin (Figure 1). Six healthy, fertile Simmental bulls, aged 3–4 years, were used for semen collection. These bulls were housed at the International Livestock Management Training Center and were confirmed to be free of reproductive issues or diseases. The study took place between April and July 2025, during which the bulls underwent routine management practices.

**FIGURE 1:**
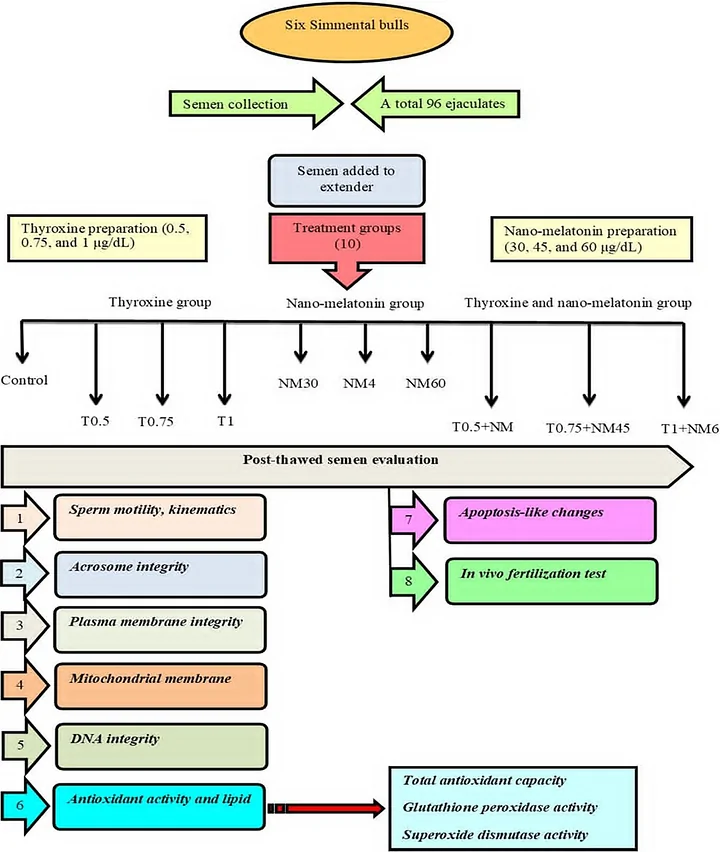
The experimental design of the study involved collecting ejaculates from six bulls and using a preparation of thyroxine and nano‐melatonin.

Over 8 weeks, a total of 96 ejaculates (16 from each bull) were collected from 6 bulls twice weekly using an artificial vagina maintained at 40–42°C. The researchers recorded the percentage of motile sperm, volume, abnormality rate, and concentration of each ejaculate. Only samples meeting the criteria of at least 75% progressive motility, a volume of 5 mL or more, an abnormal sperm count below 10%, and a sperm concentration of at least 1 × 10^9^ were selected for freezing experiments. After freezing, a portion of the straws was used for laboratory analysis. For each collection day, ejaculates from the six bulls were pooled to minimise individual bull variability. The pooled ejaculate represented one experimental replicate, and all technical measurements from multiple straws were averaged prior to statistical analysis. In contrast, the remainder was used to inseminate cows at the Governmental Station, Animal Production Farm and Agricultural Research Center.

### 2.1 Preparation of a Stock Solution of Thyroxine

Salama et al. (2025) outlined the procedure for creating a fresh supply of thyroxine. This involved dissolving 100 µg of levothyroxine sodium anhydrous (T4‐Thyro Tablets from MUP Medical Union Pharmaceuticals, Ismilia, Egypt) in 0.1 N NaOH. They adjusted the solution with distilled water until the total volume reached 100 mL, yielding a stock concentration of 100 µg/dL of thyroxine.

### 2.2 Synthesis and Characterisation Techniques of Melatonin Nanoparticles and Preparation of Stock Solution

To create melatonin nanoparticles, one gram of melatonin (Melatonin (N‐acetyl‐5‐methoxytryptamine, ≥98% purity; Sigma‐Aldrich, St. Louis, MO, USA; Cat. No. M5250) was used for the preparation of the nano‐melatonin formulation) was dissolved in 100 mL of double‐distilled water (DDW) with stirring for 3 h. This was followed by an additional hour of stirring using an ultrasonication device. The suspension was then dried at 45°C. After drying, the powder was ground for 10 h until a fine, homogeneous powder was obtained. Characterisation techniques were then used to analyse the fabricated powder (El‐Shafai et al. 2019). The grain diameter was calculated using Debye‐Scherrer's Equation 1 (El‐Shafai et al. 2023a): D = Kλ / βcosθ, where D is the calculated grain size, K is the factor value of 0.9, λ is the wavelength of the copper target, β is the diffraction peak full width at half maximum, and θ is the characteristic pattern angular position. The electrostatic stability of nanoparticles was evaluated at room temperature in DDW. The surface charges and interaction types present in the nanocomposites were analysed using diluted suspensions of the samples. An FE‐SEM instrument was used to capture micrographs of the synthesised nano‐melatonin at different magnifications. The production of nanomelatonin was confirmed by EDX analysis (Figure 2 and Figure S1).

**FIGURE 2:**
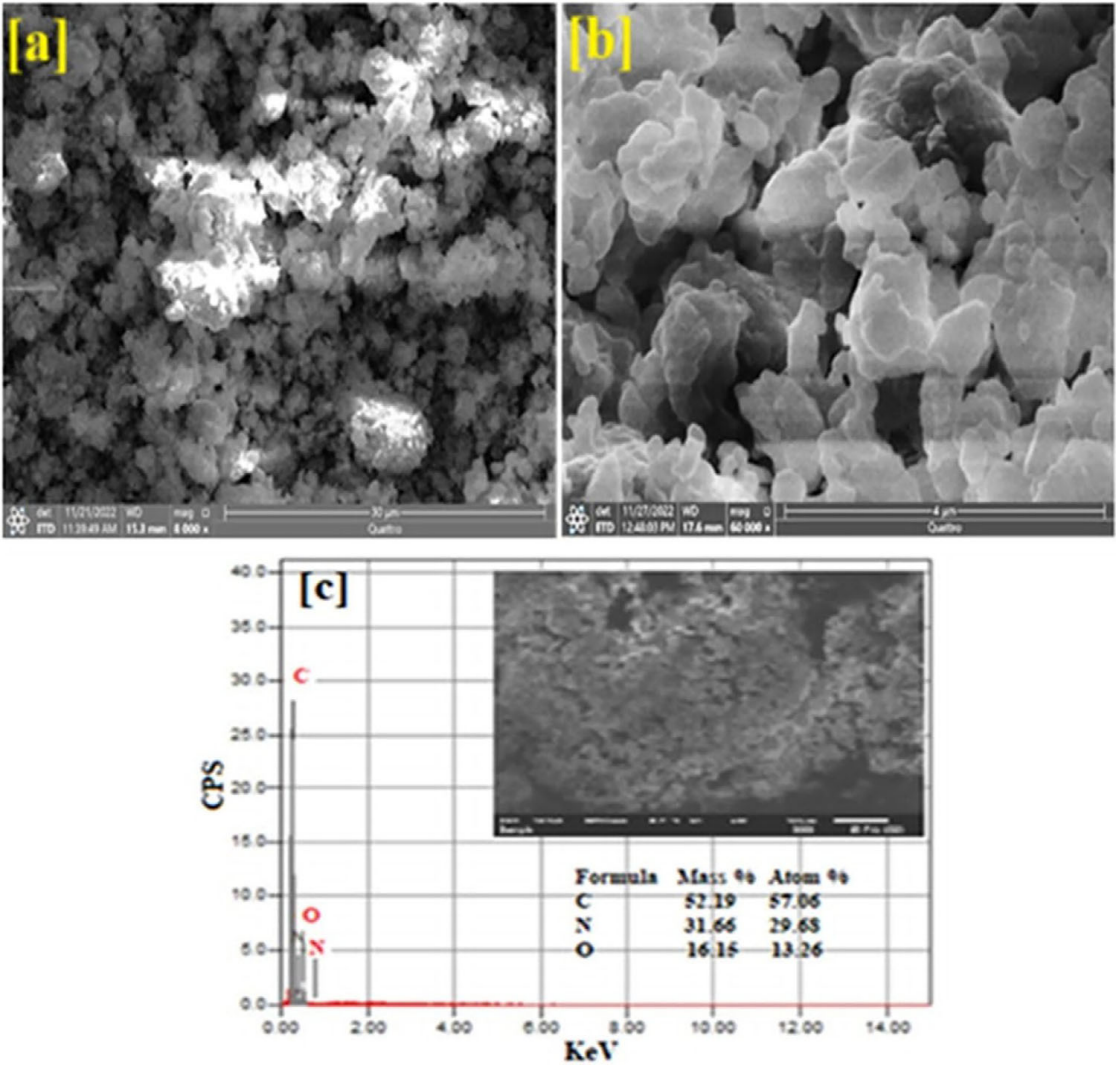
Characteristics of fabricated materials, including (a) XRD patterns of melatonin and nano‐melatonin, (b) Zeta potential of nano‐melatonin, (c) FT‐IR of nano‐melatonin and (d) Grain size of nano‐melatonin.

0.3 g of finely prepared, homogeneous powder was mixed with 100 mL of deionised water. The mixture was then subjected to ultrasonic probe treatment for 30 min at 20 kHz. The solution was then cooled to 5°C, yielding a stock concentration of 300 mg/dL of nanomelatonin (Hameed et al. 2024).

### 2.3 Extender Preparation and Experimental Design

The Tris–egg yolk–glycerol (TEYG) extender was prepared according to Leite et al. (2010). The Tris–egg yolk extender contained 24.20 g Tris(hydroxymethyl)aminomethane (Tris base), 13.60 g citric acid, 10.0 g fructose, 0.28 g penicillin, 7% (v/v) glycerol, 20% (v/v) egg yolk and double‐distilled water to a final volume of 1000 mL. The extender was prepared in a single batch and then divided into ten 100‐mL aliquots.

There were four treatment groups in this experiment. The first group served as the untreated control. The thyroxine concentrations (0.5, 0.75 and 1.0 µg/dL), designated as T0.5, T0.75 and T1, respectively, were selected based on the dose range previously reported by Salama et al. (2025), who demonstrated beneficial effects of these concentrations on post‐thaw sperm quality and fertility in buffalo bull semen. Accordingly, 500, 750, and 1000 µL of a 100 µg/dL thyroxine stock solution were added to each 100 mL of Tris–egg yolk extender to obtain the desired final concentrations.

The NM concentrations (30, 45 and 60 µg/dL), designated as NM30, NM45, and NM60, respectively, were selected based on previous reports describing the enhanced antioxidant efficiency and biological activity of nano‐melatonin at relatively low doses compared with conventional melatonin (Chuffa et al. 2021; Hameed et al. 2024). Accordingly, 10, 15 and 20 µL of an NM stock solution (300 mg/dL) were added to each 100 mL of Tris egg yolk‐based extender.

Finally, the combination groups (T0.5 + NM30, T0.75 + NM45, and T1 + NM60) were designed by pairing the corresponding thyroxine and nano‐melatonin concentrations to evaluate their potential synergistic effects on post‐thaw sperm quality, antioxidant status, apoptosis and fertility.

Sperm samples were mixed with the extender to achieve a concentration of 80 million spermatozoa per millilitre, using high‐quality ejaculates from bulls for 16 separate trials. The pooled ejaculate was divided into 10 parts and combined with various levels of thyroxine and nano‐melatonin solution. Once diluted, the samples were cooled at 4°C for 4 h to stabilise. Subsequently, the samples were transferred into 0.25 mL polyvinyl chloride straws (IMV, L'Aigle, France) and frozen at −140°C in a biofreezer (Mini Digit‐cool, ZH 400, IMV Technologies, L'Aigle, France). Samples were frozen using a programmable biofreezer at cooling rates of −30°C/min from 4°C to −15°C and −50°C/min from −15°C to −140°C before storage in liquid nitrogen. The frozen straws were thawed in a water bath at 37°C for 30 s for analysis (Dalal et al. 2018).

### 2.4 Post‐Thawed Semen Evaluation

#### 2.4.1 Sperm Motility, Kinematics, Viability and Abnormalities

Sperm motility and kinematic parameters were evaluated using a computer‐assisted sperm analysis (CASA) system (Sperm Vision, Ref. 12520/5000; Minitube GmbH, Hauptstrasse 41, 84184 Tiefenbach, Germany), following the methodology described by Dessouki et al. (2022). The system was coupled to an Olympus BX microscope (Olympus Corporation, Tokyo, Japan) equipped with a high‐speed digital camera operating at 60 frames/s under dark‐field illumination. A 5‐µL aliquot of semen was placed in a pre‐warmed Makler counting chamber and maintained at 37°C during analysis. Six randomly selected microscopic fields, comprising approximately 1,500 spermatozoa per sample, were evaluated using the manufacturer's bull‐specific CASA profile. All CASA assessments were performed by the same trained operator to minimise operator‐related variability. The evaluated sperm motion parameters included total motility (TM, %), progressive motility (PM, %), curvilinear velocity (VCL, µm/s), straight‐line velocity (VSL, µm/s), average path velocity (VAP, µm/s), straightness (STR, %), linearity (LIN, %), wobble coefficient (WOB, %) and beat cross frequency (BCF, Hz).

To evaluate viability and abnormalities, a 5‐µL drop of semen was placed on a pre‐warmed microscope slide at 40°C and mixed with an equal volume of staining solution. The staining solution consisted of 1 part 5% bluish eosin (Carl Roth GmbH + Co. KG, Karlsruhe, Germany) and 4 parts 10% aqueous nigrosin (Sigma‐Aldrich, Saint Louis, MO, United States). A glass rod was used to spread the mixture into a smear gently, and the slides were then air‐dried over a Bunsen flame. An Olympus BX‐53 light microscope (Tokyo, Japan) was used at 400× magnification to distinguish live from dead sperm cells, following the method described by Gacem et al. (2021). The same slide was used to assess sperm abnormalities, including tail abnormalities (coiled, broken) and head abnormalities (microcephalic, pear‐shaped, round, short head, loose, double head), using an oil‐immersion lens as described by Samplaski et al. (2015). A minimum of 200 spermatozoa were counted across five microscopic fields to evaluate viability and abnormalities.

#### 2.4.2 Assessment of Acrosome Integrity

The acrosome condition was evaluated using a modified Giemsa staining method, including pre‐treatment with trypan blue, as described by Santos et al. (2016). To begin, frozen‐thawed semen (200 µL) was combined with 0.2% trypan blue in a plastic tube and left to incubate in a water bath at 37°C for 10 min. The sperm were then treated with 2 mL of modified Brackett and Oliphant medium without albumin, followed by centrifugation at 700 × g for 6 min (Brackett and Oliphant 1975).

After removing the supernatant, the sperm pellet was resuspended in the same medium, and this step was repeated until the suspension turned light blue or clear. A small aliquot of the sperm suspension (20–30 µL) was then spread in a thin layer on a glass slide using another slide. The slides were dried on a heating stage at 40°C and stained with freshly prepared 10% Giemsa solution for one hour. Following rinsing with distilled water and drying, about 100 sperm cells were examined per slide under bright‐field microscopy. Intact acrosomes appeared pink or purple, while damaged ones appeared grey or white

##### 2.4.2.1 Plasma Membrane Integrity

Following the protocol outlined by Kumar et al. (2015), we conducted the hypo‐osmotic swelling test (HOST) to assess the sperm's plasma membrane function. To carry out the test, we mixed 20 µL of semen with 200 µL of a hypo‐osmotic solution at 150 mOsm/kg (0.147 g of sodium citrate and 0.2702 g of fructose dissolved in 20 mL of Milli‐Q water). The mixture was then incubated in a water bath at 37°C for 30 to 60 min. Subsequently, we observed the sample at 400x magnification using a phase‐contrast microscope on a heated slide at 38°C, covered with a coverslip, to detect sperm tail bending or coiling, which indicates intact plasma membranes.

#### 2.4.3 Mitochondrial Membrane Potential

The JC‐10 Assay for Flow Cytometry was used to evaluate mitochondrial activity in sperm cells (Salama et al. 2024). The colour of JC‐10 dye shifts from green to orange in response to changes in mitochondrial membrane potential (MMP). Thawed sperm were prepared by loading polypropylene tubes with 1 × 10^6^ sperm/mL. 500 µL of JC‐10 (C‐10 Mitochondrial Membrane Potential Assay Kit (Abcam, Cambridge, UK; Cat. No. ab112133), according to the manufacturer's instructions was combined with these semen samples, incubated for 1 h at 37°C, then washed with 1 mL of phosphate‐buffered saline (PBS) and centrifuged at 800 × g for 10 min. The resulting pellet was reconstituted in 1 mL of PBS and examined using flow cytometry. The green and orange fluorescence of the sperm populations was assessed using emission filters at 535 and 595 nm, respectively. As per Salama et al. (2024), orange fluorescence signified a higher potential, while green fluorescence indicated a lower potential for the mitochondrial membrane.

#### 2.4.4 DNA Integrity

The acridine orange test was used to assess DNA integrity following the protocol outlined by Ribas‐Maynou et al. (2013). Semen samples were placed on glass slides and fixed in Carnoy's solution (glacial acetic acid:methanol, 1:3) for 12 h. After drying, the slides were immersed in a tampon solution (80 mmol/L citric acid, 15 mmol/L Na_2_HPO_4_, and 15 mmol/L Na_2_HPO_4_, pH 2.5) at 75°C for 5 min. Subsequently, the slides were stained with a 0.2 mg/mL acridine orange solution (Sigma‐Aldrich, St. Louis, MO, USA; Cat. No. A9231). After rinsing the slides with water, they were covered and examined using an epifluorescence microscope. One hundred cells were analysed from each semen sample. Cells with normal DNA fluoresced green, while those with aberrant DNA exhibited fluorescence in a range of colours, from red to yellow‐green (Salama et al. 2024).

#### 2.4.5 Antioxidant Activity and Lipid Peroxidation

Four straws of frozen–thawed semen from each group were centrifuged at 1000 × g for 10 min, and the resulting supernatants were collected for biochemical analyses. Total antioxidant capacity (TAC) was determined using the Antioxidant Assay Kit (Cayman Chemical, Ann Arbor, MI, USA; Cat. No. 709001). Glutathione peroxidase (GPx) activity was measured using the Glutathione Peroxidase Assay Kit (Cayman Chemical, Ann Arbor, MI, USA; Cat. No. 703102). Superoxide dismutase (SOD) activity was determined using the Superoxide Dismutase Assay Kit (Cayman Chemical, Ann Arbor, MI, USA; Cat. No. 706002). Malondialdehyde (MDA) levels were quantified using the Lipid Peroxidation (MDA) Assay Kit (Cayman Chemical, Ann Arbor, MI, USA; Cat. No. 700870). All assays were performed in quadruplicate according to the manufacturer's instructions.

##### 2.4.5.1 Total Antioxidant Capacity

According to Lone et al. (2016), an assay kit from Michigan, USA, was used to measure the total antioxidant capacity. Initially, 300 µL of chromogen, 20 µL of metmyoglobin and 20 µL of sample or standard were added to each well of the plate in duplicate. Subsequently, 80 µL of H_2_O_2_ was added to the plate to initiate the reaction. The plate was then closed and allowed to sit at room temperature for 5 min. A plate reader was used to measure absorbance at 750 nm, and the TAC (mm) was determined according to Lone et al. (2016) using the equation of the standard curve derived by linear regression.

##### 2.4.5.2 Glutathione Peroxidase Activity

The GPx test kit was used to measure glutathione peroxidase activity (Kumar et al. 2015). Briefly, 20 µL of standards or samples, 1000 µL of assay buffer, 50 µL of the co‐substrate mixture, and 20 µL of cumene hydroperoxide were added to each well of the plate to start the reaction. The absorbance at 340 nm was measured every minute for at least 5 min using a plate reader. Subsequently, the GPx standard curve was generated, and GPx activity (nmol/min/mL) for each sample was calculated.

##### 2.4.5.3 Superoxide Dismutase Activity

The SOD test kit was used to measure superoxide dismutase activity according to the method outlined by Kumar et al. (2015). In brief, 20 µL of diluted xanthine oxidase, 200 µL of the diluted radical detector, and 10 µL of standards or samples were added to each well of the plate. The plate was gently shaken and left at room temperature for 20 min. Afterwards, the SOD activity (U/mL) of each sample was determined by establishing an SOD standard curve.

##### 2.4.5.4 Malondialdehyde Content

The TBARS test kit was used to measure malondialdehyde levels (Kumar et al. 2015). To begin, a clean test tube was filled with 50 µL of a sodium dodecyl sulphate solution, 50 µL of the sample or standard and 200 µL of the colour reagent. After allowing the test tubes to cool on ice for 10 min, they were placed in a hot‐water bath for 60 min. Following this, the mixture was centrifuged for 10 min at 4°C at 1600 × g. Subsequently, a colorimetric plate was filled with 40 µL of the centrifuged mixture, and the absorbance at 535 nm was recorded. By constructing the MDA standard curve, the MDA concentration of the samples (µM/mL) was determined.

#### 2.4.6 Apoptosis‐Like Changes

Apoptotic changes in spermatozoa were evaluated using the Phosphatidylserine Detection Kit (Annexin V‐FITC/Propidium Iodide; IQ Products BV, Groningen, The Netherlands; Cat. No. IQP‐116F), according to the manufacturer's instructions. The assay detects phosphatidylserine (PS) externalisation as an early marker of apoptosis. Following washing with calcium buffer, sperm samples were incubated with 10 µL Annexin V‐FITC for 20 min on ice in the dark. Subsequently, 10 µL propidium iodide (PI) was added, and the samples were incubated for an additional 10 min on ice in the dark. Flow cytometric analysis was performed using an Accuri C6 Flow Cytometer (BD Accuri C6, BD Biosciences, San Jose, CA, USA) equipped with 488‐nm blue and 640‐nm red lasers and operated using BD Accuri C6 Software version 1.0.264.21. For each sample, at least 10,000 sperm events were acquired at a low flow rate. Spermatozoa were first identified and gated according to their forward‐scatter (FSC) and side‐scatter (SSC) characteristics to exclude debris and non‐sperm particles. Annexin V‐FITC fluorescence was detected in the FL1 channel (533/30 nm), whereas propidium iodide (PI) fluorescence was detected in the FL3 channel (>670 nm long‐pass filter). Instrument settings and fluorescence compensation were optimised using unstained and single‐stained controls before sample acquisition. Based on Annexin V‐FITC/PI staining, spermatozoa were classified into four subpopulations as described by Masoudi et al. (2018): viable non‐apoptotic sperm (Annexin V−/PI−), early apoptotic sperm (Annexin V+/PI−), late apoptotic sperm (Annexin V+/PI+) and necrotic sperm (Annexin V−/PI+). Data were analysed using BD Accuri C6 software

### 2.5 In Vivo Fertilization Test

One hundred and forty healthy cyclic cows were divided into four groups (35 cows per group) for this study. On Day 7, they were administered a single injection of 750 µg loprostenol sodium (Estrumate from Coopers Animal Health Ltd., Berkhamsted, England) and two injections of 20 µg Buserelin acetate (Receptal, Intervet International, Netherlands) on Days 0 and 9 for GPG synchronisation (Figure S2).

Based on the results of the in vitro semen quality assessment, the combination of 0.5 µg/dL thyroxine (T0.5) and 30 µg/dL nano‐melatonin (NM30) was selected for the fertility trial because it produced the most favourable overall post‐thaw sperm quality, antioxidant status, and apoptotic profile. Therefore, only this optimised combination was evaluated under field conditions and compared with the control, T0.5 and NM30 groups. This approach minimised the number of animals required and reduced the logistical and economic constraints associated with large‐scale artificial insemination trials while allowing validation of the most promising treatment under practical conditions. Two hundred and eighty frozen‐thawed straws containing 20 × 106 spermatozoa from four different extenders (Control, T0.5, NM30 and T0.5 + NM30) were used for artificial insemination on Day 10 for all cows with duplicate inseminations (morning and evening). In August 2025, the insemination operations were conducted. Pregnancy was verified in all inseminated cows on Day 45 following insemination using linear probe ultrasonography with a real‐time B‐mode veterinary ultrasound instrument (Esaote Pie Medical Aquila Pro‐Vet with 6.0/8.0 MHz LA Rectal Veterinary, Esaote, Italy).

### 2.6 Statistical Analysis

Data were analysed using SAS software (Version XX, SAS Institute Inc., Cary, NC, USA). Data normality and homogeneity of variance were verified using Shapiro–Wilk and Levene's tests, respectively. Differences among groups were analysed using one‐way ANOVA followed by Tukey's post hoc test. Data are presented as mean ± SEM. Pregnancy rates were analysed using the Chi‐square test. Differences were considered statistically significant at *p* < 0.05.

## 3 Results

### 3.1 Characterisation of Nano‐Melatonin

The characteristic patterns of fabricated melatonin and nano‐melatonin are shown in Figure 2a. The crystal structure of melatonin appeared at 2θ angles of 10.8°, 14.5°, 16°, 18.6°, 20.2°, 22.4°, 23.9°, 24.7°, 25.7°, 28°, 31°, 38.4°, 64° and 77.5° due to the crystal structure of melatonin. Nano‐melatonin exhibited patterns at 10.6°, 14.4°, 16°, 18.7°, 20.3°, 22°, 24°, 24.7°, 25.7°, 27.9°, 30.5°, 44°, 64° and 77.7°. Zeta values were recorded at −39.5 mV (Figure 2b). The grain size of melatonin was determined to be 72 nm, while nano‐melatonin had an average diameter of 22 nm (Figure 2d).

The vibrational bands of nano‐melatonin were detected (Figure 2c). The OH group appeared at 3304 cm^−1^, the NH group at 3075 cm^−1^, and 2920 cm^−1^ was attributed to the aliphatic CH group. Functional groups containing oxygen were identified at 1645 cm^−1^ for carboxyl (C═O), 1564 cm^−1^ and 1482 cm^−1^ for carboxyl (C═C), 1215 cm^−1^ for epoxy (C─O), 1051 cm^−1^ for alkoxy (C─O), and 918 cm^−1^ for carboxyl (C─OH) (El‐Shafai et al. 2023b). The presence of these functional groups in melatonin emphasises the formation of nano‐melatonin.

In Figures 3a and 3b, the nano‐melatonin is depicted as a spherical structure with a non‐smooth surface and high‐purity crystals. Different magnifications were utilised to clarify the shape and distribution of the particles. The components and purity of the nanomelatonin were confirmed by EDX analysis, with the elemental percentages shown in Figure 3c.

**FIGURE 3:**
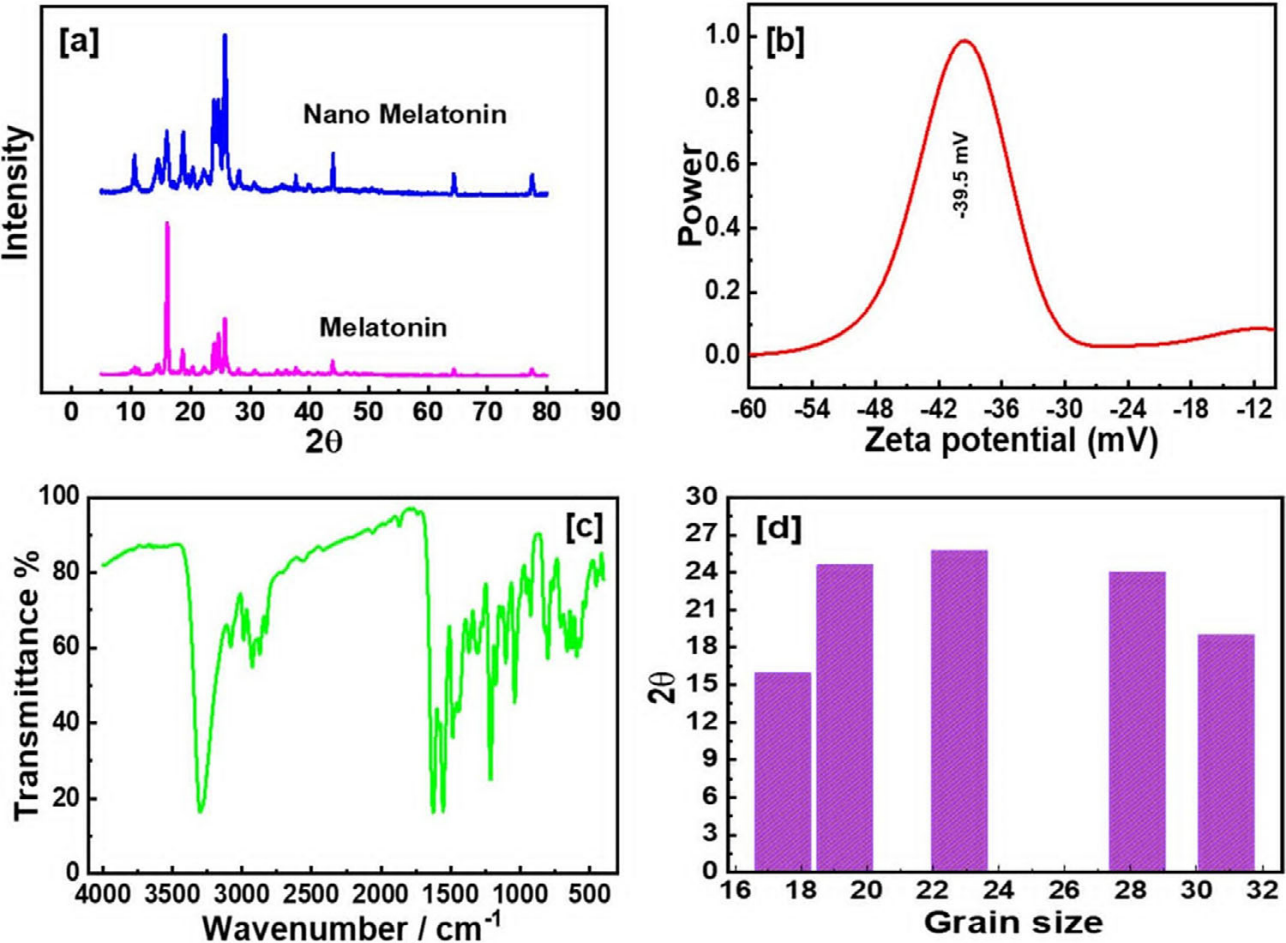
SEM micrographs of nano‐melatonin at different magnifications (a and b), and EDX analysis of nano‐melatonin components (c).

### 3.2 Effects of Thyroxine, Nano‐Melatonin, and a Combination of Thyroxine and Nano‐Melatonin Groups on the Motility and Kinematics of Frozen–Thawed Sperm

The treatments in all tables can be divided into four main groups: control, thyroxine, nano‐melatonin, and a combination of thyroxine and nano‐melatonin groups. The results presented in Table 1 show a significant increase (p < 0.05) in motility and in speed values (VAP, VCL and VSL, µm/s) in the thyroxine‐mixed nano‐melatonin group (T0.5 + NM30) compared to the other groups. Following closely was the nano‐melatonin group (NM30). On the other hand, the control group exhibited lower speed values, with no significant differences compared to the thyroxine, nano‐melatonin, and mixed groups (T0.75, NM60 and T1 + NM60). Furthermore, the STR, LIN, and BCF (Hz) values in the T0.5 + NM30 group were significantly higher (p < 0.05) than those in the control and other groups. There were no significant differences (p > 0.05) among all groups in terms of WOB, as shown in Table 1.

**TABLE 1:** Effect of different T4 and nano‐melatonin additives on motility and kinematic parameters of post‐thawed bull sperm.

Parameters/Groups	Control	T0.5	T0.75	T1	NM30	NM45	NM60	T0.5 + NM30	T0.75 + NM45	T1 + NM60
Total motility (%)	41.23 ± 0.49^e^	47.62 ± 0.72^d^	40.69 ± 0.47^e^	40.92 ± 0.56^e^	54.85 ± 0.75^b^	51.46 ± 0.74^c^	42.77 ± 0.67^e^	58.31 ± 0.99 ^a^	51.92 ± 0.64^c^	45.77 ± 0.77^d^
Progressive motility (%)	36.54 ± 0.64^f^	41.85 ± 0.72^d^	34.62 ± 0.57^g^	34.00 ± 0.66^g^	49.23 ± 0.65^b^	45.31 ± 0.77^c^	36.77 ± 0.61^f^	52.38 ± 0.54^a^	46.31 ± 0.67^c^	39.46 ± 0.53^e^
VAP (µm/s)	61.23 ± 0.81^ef^	65.92 ± 0.76^d^	59.85 ± 0.81^f^	59.69 ± 0.90^f^	70.00 ± 0.53^b^	67.23 ± 0.80^cd^	61.85 ± 0.82^ef^	73.38 ± 0.68^a^	68.31 ± 0.69^bc^	63.15 ± 0.78^e^
VCL (µm/s)	76.85 ± 0.59^e^	80.38 ± 0.79^d^	75.46 ± 0.46^ef^	74.31 ± 0.56^f^	84.77 ± 0.89^b^	81.54 ± 0.58^cd^	76.46 ± 1.05^e^	88.62 ± 0.79^a^	82.85 ± 0.68^bc^	77.23 ± 0.66^e^
VSL (µm/s)	18.31 ± 0.61^d^	21.08 ± 0.47^c^	17.92 ± 0.60^d^	17.54 ± 0.48^d^	23.00 ± 0.63^b^	21.31 ± 0.49^c^	17.92 ± 0.54^d^	26.15 ± 0.44^a^	21.62 ± 0.45^bc^	19.00 ± 0.53^d^
STR (%)	30.02 ± 1.20^bc^	32.00 ± 0.72^bc^	30.08 ± 1.23^bc^	29.54 ± 1.11^c^	32.85 ± 0.86^b^	31.80 ± 0.96^bc^	29.10 ± 1.09^c^	35.69 ± 0.75^a^	31.69 ± 0.76^bc^	30.12 ± 0.86^bc^
LIN (%)	23.82 ± 0.76^d^	26.27 ± 0.71^bc^	23.75 ± 0.79^d^	23.64 ± 0.72^d^	27.15 ± 0.76^b^	26.16 ± 0.68^bc^	23.53 ± 0.87^d^	29.56 ± 0.63^a^	26.14 ± 0.67^bc^	24.64 ± 0.76^cd^
WOB (%)	79.78 ± 1.42^a^	82.05 ± 0.89^a^	79.35 ± 1.19^a^	80.39 ± 1.37^a^	82.66 ± 0.89^a^	82.50 ± 1.11^a^	81.09 ± 1.64^a^	82.89 ± 1.04^a^	82.55 ± 1.24^a^	81.81 ± 1.01^a^
BCF (Hz)	28.62 ± 0.93^e^	31.31 ± 0.93^cd^	26.46 ± 0.69^f^	25.38 ± 0.73^f^	34.92 ± 0.70^ab^	33.23 ± 0.63^bc^	30.31 ± 0.83^de^	36.85 ± 0.72^a^	33.00 ± 0.79^bc^	29.54 ± 0.63^de^

Number of ejaculates = 80. The means bearing one common superscript within the same row were similar (P *≥* 0.05).

Abbreviations: BCF, beat cross frequency (Hz); LIN, linearity (VSL/VCL; %); STR, straightness (VSL/VAP; %); VAP, average pathway velocity (µms); VCL, curvilinear velocity (µms); VSL, straight‐line velocity (µms); WOB, wobble (VAP/VCL; %).

### 3.3 Effects of Thyroxine, Nano‐Melatonin and a Combination of Thyroxine and Nano‐Melatonin Groups on the Viability, Abnormality and Function Parameters of Frozen‐Thawed Sperm

According to the findings in Table 2, the group treated with T0.5 + NM30 showed significantly higher (p < 0.05) viability, acrosome integrity, plasma membrane integrity and mitochondrial membrane potential than the other groups in the frozen‐thawed semen. Regarding DNA integrity, the T0.5, NM30 and T0.5 + NM30 groups showed significantly higher levels (p < 0.05) than the other groups. There were no significant differences (p > 0.05) in abnormality percentages across all groups (Table 2).

**TABLE 2:** Effect of different T4 and nano‐melatonin additives on viability, abnormality and function parameters of frozen‐thawed bull sperm.

Parameters/Groups	Control	T0.5	T0.75	T1	NM30	NM45	NM60	T0.5 + NM30	T0.75 + NM45	T1 + NM60
Viability (%)	43.62 ± 0.70^ef^	49.38 ± 0.67^d^	42.54 ± 0.62^f^	41.77 ± 0.58^f^	56.92 ± 0.81^b^	53.23 ± 0.74^c^	45.15 ± 0.74^e^	60.69 ± 0.90^a^	53.69 ± 0.60^c^	47.62 ± 0.85^d^
Abnormality (%)	14.08 ± 0.30^a^	13.77 ± 0.26^a^	13.92 ± 0.27^a^	14.23 ± 0.28^a^	14.00 ± 0.28^a^	14.31 ± 0.29^a^	14.00 ± 0.29^a^	13.38 ± 0.33^a^	13.69 ± 0.26^a^	13.85 ± 0.32^a^
Acrosome integrity (%)	65.77 ± 0.89^ef^	69.15 ± 0.71^cd^	63.92 ± 0.59^fg^	62.62 ± 0.47^g^	72.00 ± 0.62^b^	70.69 ± 0.51^bc^	67.31 ± 0.74^de^	75.69 ± 0.93^a^	71.31 ± 0.61^b^	67.77 ± 0.66^de^
Membrane integrity (%)	47.31 ± 0.69^fg^	50.00 ± 0.77^cde^	45.54 ± 0.57^gh^	44.46 ± 0.65^h^	53.08 ± 0.67^b^	50.85 ± 0.62^cd^	48.77 ± 0.62^ef^	57.23 ± 0.86^a^	51.69 ± 0.72^bc^	48.92 ± 0.51^def^
MMP (%)	49.08 ± 0.57^de^	54.62 ± 0.63^bc^	47.85 ± 0.68^ef^	46.69 ± 0.51^f^	55.38 ± 0.67^b^	53.69 ± 0.64^bc^	50.23 ± 0.59^d^	58.77 ± 0.81^a^	52.92 ± 0.63^c^	50.15 ± 0.62^d^
DNA integrity (%)	86.54 ± 1.06^de^	89.92 ± 0.53^abc^	85.15± 0.88^ef^	84.08 ± 0.84^f^	90.69 ± 0.74^ab^	89.38 ± 0.62^bc^	87.85 ± 0.68^cd^	92.00 ± 0.67^a^	88.77 ± 1.05^bcd^	86.85 ± 0.76^de^

Number of ejaculates = 80. The means bearing one common superscript within the same row were similar (P *≥* 0.05).

Abbreviation: MMP, mitochondrial membrane potential.

### 3.4 Effects of Thyroxine, Nano‐Melatonin and a Combination of Thyroxine and Nano‐Melatonin Groups on Antioxidant Parameters of Post‐Thawed Sperm

Only the group treated with thyroxine at 0.5 µg/dL (T0.5) and all groups treated with nano‐melatonin, including the combination of thyroxine and nano‐melatonin groups, especially the T0.5 + NM30 group, showed improvements in antioxidant parameters (p < 0.05). These improvements were observed in GPx, SOD and TAC values, with lower MDA levels than in the control group. The only exceptions were the T0.75 and T1 groups within the thyroxine‐treated groups, which did not exhibit significant differences (p > 0.05) in antioxidant parameter levels (see Table 3).

**TABLE 3:** Effect of different T4 and nano‐melatonin additives on antioxidant parameters of post‐thawed bull sperm.

Parameters/Groups	Control	T0.5	T0.75	T1	NM30	NM45	NM60	T0.5 + NM30	T0.75 + NM45	T1 + NM60
GPx (nmol/min/mL)	14.85 ± 0.33^f^	16.62 ± 0.39^e^	14.38 ± 0.28^f^	14.00 ± 0.28^f^	19.08 ± 0.49^bc^	18.23 ± 0.53^cd^	17.77 ± 0.46^de^	21.31 ± 0.54^a^	19.92 ± 0.38^b^	17.38 ± 0.46^de^
SOD (U/mL)	33.31± 0.65^d^	36.46 ± 0.84^c^	31.85 ± 0.51^d^	30.92 ± 0.49^d^	40.00 ± 0.54^b^	38.69 ± 0.39^b^	36.77 ± 0.53^c^	42.08 ± 0.68^a^	40.23 ± 0.52^b^	38.54 ± 0.43^b^
TAC (Mm)	1.39 ± 0.02^f^	1.64 ± 0.03^e^	1.37 ± 0.02^f^	1.36 ± 0.01^f^	1.90 ± 0.01^c^	1.76 ± 0.02^d^	1.66 ± 0.02^e^	2.25 ± 0.04^a^	2.00 ± 0.05^b^	1.65 ± 0.01^e^
MDA (µM/mL)	1.29 ± 0.01^a^	1.01 ± 0.02^b^	1.31 ± 0.0^a^	1.33 ± 0.01^a^	0.77 ± 0.02^e^	0.87 ± 0.02^cd^	0.92 ± 0.02^c^	0.61 ± 0.02^f^	0.84 ± 0.01^d^	0.98 ± 0.02^b^

Number of ejaculates = 80. The means bearing one common superscript within the same row were similar (*p ≥* 0.05).

Abbreviations: GPx, glutathione peroxidase; SOD, superoxide dismutase; TAC, total antioxidant capacity; MDA, malondialdehyde.

### 3.5 Effects of Thyroxine, Nano‐Melatonin and a Combination of Thyroxine and Nano‐Melatonin Groups on Apoptosis‐Like Changes in Post‐Thawed Sperm

According to Table 4, the T0.5 + NM30 group had the highest viable sperm cell count (p < 0.05) compared to the other groups. Conversely, the control, T0.75, and T1 groups had the lowest levels of early apoptotic sperm cells compared to the other groups (p < 0.05). Regarding late apoptotic sperm cells, the group treated with thyroxine at 0.5 µg/dL (T0.5) and all groups treated with nano‐melatonin, as well as the combination of thyroxine and nano‐melatonin groups, displayed significantly lower levels than the control group (p < 0.05). Furthermore, the T0.5, NM30, NM45, T0.5 + NM30 and T0.75 + NM45 groups decreased the amount of necrotic sperm cells compared to the control group and other groups (p < 0.05), as shown in Table 4.

**TABLE 4:** Effect of different T4 and nano‐melatonin additives on apoptotic‐like changes of post‐thawed bull sperm (Annexin V/PI assay).

Parameters /Groups	Control	T0.5	T0.75	T1	NM30	NM45	NM60	T0.5 + NM30	T0.75 + NM45	T1 + NM60
Viable (A‐/PI‐)	45.46 ± 0.70^fg^	52.31 ± 0.86^d^	44.31 ± 0.63^g^	43.62 ± 0.60^g^	58.15 ± 0.59^b^	54.92 ± 0.74^c^	46.54 ± 0.53^ef^	63.00 ± 0.85^a^	57.38 ± 0.81^b^	48.38 ± 0.85^e^
Early Apoptotic (A+/PI‐)	6.23 ± 0.30^d^	11.85 ± 0.37^b^	5.77 ± 0.26^d^	5.46 ± 0.22^d^	13.23 ± 0.52^a^	11.92 ± 0.39^b^	11.62 ± 0.38^b^	10.15 ± 0.37^c^	11.85 ± 0.36^b^	12.23 ± 0.43^ab^
Late Apoptotic (A+/PI+)	29.92 ± 0.47^a^	23.38 ± 0.79^b^	30.85 ± 0.65^a^	31.38 ± 0.76^a^	20.08 ± 0.42^c^	22.62 ± 0.45^b^	24.15 ± 0.47^b^	18.46 ± 0.37^c^	19.38 ± 0.42^c^	22.54 ± 0.68^b^
Necrotic (A‐/PI+)	18.38 ± 0.69^a^	12.46 ± 1.32^b^	19.08 ± 1.30^a^	19.54 ± 0.93^a^	8.54 ± 0.81^c^	10.54 ± 1.12^bc^	17.69 ± 0.62^a^	8.39 ± 0.73^c^	11.38 ± 1.08^bc^	16.85 ± 1.17^a^

Number of ejaculates = 80. The means bearing one common superscript within the same row were similar (p *≥* 0.05).

Annexin V/PI assay (Annexin V and propidium iodide (PI) labelling of cells).

### 3.6 Effects of Thyroxine, Nano‐Melatonin and a Combination of Thyroxine and Nano‐Melatonin Groups on the Fertility of Post‐Thawed Sperm

Figure 4 shows a significant increase in conception rates (*p* < 0.05) for the NM30 group (77.14%) and the T0.5 + NM30 group (82.86%) compared to the control group (48.57%) and the T0.5 group (57.14%).

**FIGURE 4:**
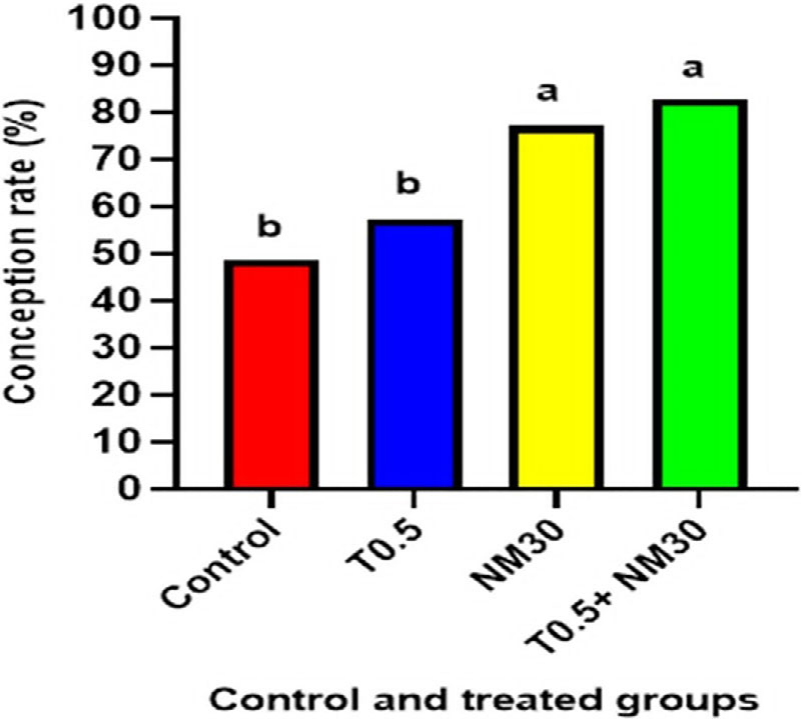
Conception rates of control, T0.5, NM30, and T0.5 + NM30 groups. Each group consists of 35 cows.

## 4 Discussion

Altering melatonin into nano‐sized materials shows superior effectiveness compared to free melatonin, making it functionally significant for clinical intervention (Chuffa et al. 2021). Melatonin is prone to degradation when exposed to air and light and is poorly soluble in water. By encapsulating melatonin in nanoparticles, degradation can be prevented, photostability can be increased (Tursilli et al. 2006), and long‐term stability can be achieved even after 7 months of storage at 4°C (Hafner et al. 2011). Our results indicated good stability of nanomelatonin in solution, which may be due to the crystal structure of melatonin observed at 2θ between 10.8° and 77.5°. In contrast, nano‐melatonin exhibited patterns between 10.6° and 77.7°. These results indicate the stability of melatonin's crystal structure in both bulk and nanocrystal forms. However, changes in the positions of the characteristic angles and the pattern intensities are due to modifications in the binding length within the nanocrystal. Despite these changes, the distribution of atoms in the nanocrystal remained consistent with that of the bulk crystal. The stability of nano‐melatonin in the solution may also be related to the Zeta values, which were recorded at −39.5 mV (Figure 2b). Zeta values are used to measure stability, with values of ±30 mV suggesting moderate stability, values of ±60 mV indicating good dispersion stability, and values exceeding ±60 mV indicating excellent stability (El‐Wakeil et al. 2024).

The size of nanoparticles plays an important role in determining their effectiveness. In this study, the average diameter of melatonin nanoparticles is small, at 22 nm, which could enhance the properties of cryopreserved sperm. This finding is consistent with Choi and Jang (2022), who observed that nanoparticles, molecules with a diameter of less than 100 nm, can be beneficial in preserving reproductive cells during cryopreservation due to their unique chemical and physical characteristics. As a result, we administered significantly lower doses of nano‐melatonin, following the findings of Tursilli et al. (2006), compared to the typical melatonin doses used in semen supplementation. Specifically, we used doses of 30, 45, and 60 µg/dL of nanomelatonin, whereas Niu et al. (2024) used doses of 0.1, 0.3, 0.5 and 0.7 mg/mL of melatonin. This indicates that even a lower dose of nano‐melatonin can be highly effective (Hameed et al. 2024). This study may be the first to investigate the protective effects of nano‐melatonin and thyroxine on semen cryopreservation in Simmental bulls.

The results of this study suggest that a combination of thyroxine and nano‐melatonin (T0.5 + NM30) is more effective than the control and other groups in enhancing sperm motility, viability, BCF (Hz), and speed values (VAP, VCL, and VSL, µm/s). This increase in sperm motility may be attributed to thyroid hormones that do not require gene involvement. This occurs when hormones attach to receptors located outside the cell nucleus in different areas of the sperm cell. This interaction triggers the production of cyclic adenosine monophosphate and the release of calcium (Ca^2+^), ultimately leading to increased sperm motility (La Vignera et al. 2017). Studies have shown that thyroxine can rapidly improve flagellar motility in sperm cells, leading to hyperactivity (Mendeluk and Rosales 2016). This increase in sperm motility may also be due to melatonin's interaction with calmodulin (Benítez‐King and Antón‐Tay 1993), which affects the cytoskeletal elements of sperm and enhances motility by stimulating the cellular influx of Ca^2^^+^ (Huerto‐Delgadillo et al. 1994). Sperm motility and velocity are enhanced by calcium and its channels, which regulate the transition from symmetric to asymmetric flagella wave patterns (Si 1997).

Additionally, the combination of thyroxine and nano‐melatonin (T0.5 + NM30) is more effective in improving the functional parameters of frozen‐thawed bull sperm compared to the control and other groups. This enhanced effectiveness may be attributed to thyroxine, which improves mitochondrial activity, as suggested by Condorelli et al. (2019). The improvement in mitochondrial activity observed in this study could be linked to enhanced oxidative phosphorylation. This process boosts sperm motility via a specific biochemical pathway until all available substrates are depleted. Additionally, the attachment of thyroxine to cellular DNA can increase both mitochondrial quantity and function, thereby enhancing basal metabolic rate, as noted by Oppenheimer and Tavernetti (1962). Conversely, high doses of thyroxine have been shown to inhibit cytochrome c oxidase activity and reduce mitochondrial function, as reported by Romano et al. (2017). As well as this, the improvement of sperm functional parameters in frozen‐thawed bull sperm may also be related to nano‐melatonin. Melatonin functions by removing harmful reactive nitrogen and reactive oxygen species, blocking the opening of the mitochondrial permeability transition pore, and regulating the activity of various stress‐responsive genes and antioxidant enzymes, as reported by Gualtieri et al. (2021).

Additionally, the combination of thyroxine and nano‐melatonin (T0.5 + NM30) is more effective in improving antioxidant parameters and decreasing lipid peroxidase levels of frozen‐thawed bull sperm compared to the control and other groups. This improvement may be related to thyroxine, as reported by Salama et al. (2025). Our research revealed that nano‐melatonin improves antioxidant properties in semen, consistent with the findings of Rao et al. (2015), who observed that nanoparticles can now perform more specialised functions, such as providing antioxidant benefits during the cryopreservation of human somatic cells. The advancement may also be attributed to nano‐melatonin's capability to protect against oxidative stress through various mechanisms, including boosting antioxidant enzyme activity, neutralising free radicals (Li et al. 2022), and activating signalling pathways such as SIRT‐1/FOXO‐1 and Nrf‐2/HO‐1 (Kumar et al. 2021). These pathways are essential in regulating antioxidant proteins.

This research has revealed that the combination of thyroxine and nano‐melatonin (T0.5 + NM30) is more effective at improving viability and reducing late‐apoptotic and necrotic sperm cells. This improvement may be attributed to thyroxine, which enhances mitochondrial membrane potential, as reported by Salama et al. (2025). On the other hand, mitochondrial malfunction may cause sperm cells to undergo apoptosis, reducing their ability to fertilise, as noted by Khalil et al. (2023). Nano‐melatonin also possesses anti‐apoptotic properties, possibly due to its ability to suppress cytochrome c release and caspase‐3 activation, as well as its influence on the expression of the pro‐apoptotic Bax and the anti‐apoptotic Bcl‐2. The JAK2/STAT3 pathway mediates melatonin‐induced Bax/Bcl‐2 translocation, while p38 MAPK inhibition and ERK activation contribute to its anti‐apoptotic effects, as explained by Yang et al. (2013). Furthermore, activation of specific histone deacetylases via SIRT1/PPAR coactivator (PGC‐1α) and AMPK/SIRT3/SOD2 pathways is another aspect of melatonin‐activated signalling cascades, as described by Carbone et al. (2020).

The nano‐melatonin (NM30) or thyroxine‐mixed nano‐melatonin (T0.5 + NM30) groups have been shown to improve fertility. This improvement may be attributed to melatonin's ability to enhance sperm motility, functionality, and antioxidant activity, while also reducing lipid peroxidation and decreasing apoptosis (Makris et al. 2023). On the other hand, thyroxine may affect mitochondrial function, as Salama et al. (2025) reported that adding thyroxine to semen can enhance mitochondrial membrane potential, thereby increasing mitochondrial ATP production. Given the well‐established connection between MMP and sperm motility, this is crucial for sperm viability and fertilisation capacity (Martinez‐Pastor et al. 2004). According to Eliasson (2010), these factors are essential in determining the likelihood of sperm fertilisation. This study has some limitations, including the use of pooled semen samples, the absence of molecular validation of signalling pathways, and limited nanoparticle characterisation. Future studies should investigate the molecular mechanisms underlying the synergistic effects of thyroxine and nano‐melatonin

## 5 Conclusion

In conclusion, supplementation of semen extenders with thyroxine and nano‐melatonin, particularly the T0.5 + NM30 combination, significantly improved post‐thaw sperm quality, antioxidant defence and fertility of Simmental bull semen. These beneficial effects are likely attributable to the complementary biological actions of the two molecules. Thyroxine enhances sperm energy metabolism and mitochondrial activity, promoting ATP production and improving sperm motility, whereas nano‐melatonin protects spermatozoa against cryopreservation‐induced oxidative stress through its potent antioxidant and anti‐apoptotic properties, thereby preserving plasma membrane integrity and mitochondrial function. The synergistic interaction between these mechanisms likely underlies the superior post‐thaw sperm quality, enhanced antioxidant defence and improved fertility observed with the combined treatment. These findings highlight the potential of combining thyroxine and nano‐melatonin as an effective strategy to optimise bull semen cryopreservation and improve reproductive efficiency in artificial insemination programmes.

## Author Contributions

M. A. A., A. H. A., M. S. S. and M. S. contributed to the conceptualisation and design of the study. M. A. A., M. S. S., M. A. A., S. M. S., A. M. S., Z. S. I., M. S. and A. A. performed the experiments, sample collection and laboratory analyses. N. M. E. contributed to the synthesis and characterisation of nanoparticles. A. M. S. and A. O. A. performed statistical analysis and data interpretation. S. M., R. Y. E. and O. H. A. contributed to technical support, validation and resources. M. S., A. K., E. E., E. A. A. and K. A. K. supervised the work, interpreted the data, and revised the manuscript critically for important intellectual content. All authors contributed to manuscript preparation, reviewed the final version and approved the manuscript for publication.

## Funding

This work was supported by the Deanship of Scientific Research, Vice Presidency for Graduate Research, King Faisal University, Saudi Arabia (Grant No. KFU263905).

## Ethics Statement

The authors confirm that the ethical policies of the journal, as noted on the journal's author guidelines page, have been adhered to. All experimental procedures involving animals were approved by the Institutional Animal Care and Use Committee (IACUC) of the Faculty of Veterinary Medicine, Kafrelsheikh University, Egypt (Approval No. KFS.190‐122). The study was conducted in accordance with institutional guidelines for the care and use of experimental animals and complied with the ARRIVE guidelines (Animal Research: Reporting of In Vivo Experiments).

## Conflicts of Interest

The authors declare no conflicts of interest.

## Supporting information




**Supporting File 1**: vms371252‐sup‐0001‐SuppMat.pdf

## Data Availability

Data is available upon request from the corresponding author.
