# Supplementary material for: Synergistic Effects of Thyroxine and Nano‐Melatonin on Post‐Thaw Sperm Quality, Antioxidant Defence and Fertility of Cryopreserved Simmental Bull Semen
Source: Vet Med Sci. 2026 Sep 30;12(6):e71252. doi: 10.1002/vms3.71252 (PMC13627263; doi:10.1002/vms3.71252)
Supplement: Supplementary file 1 [file VMS3-12-e71252-s001.pdf]

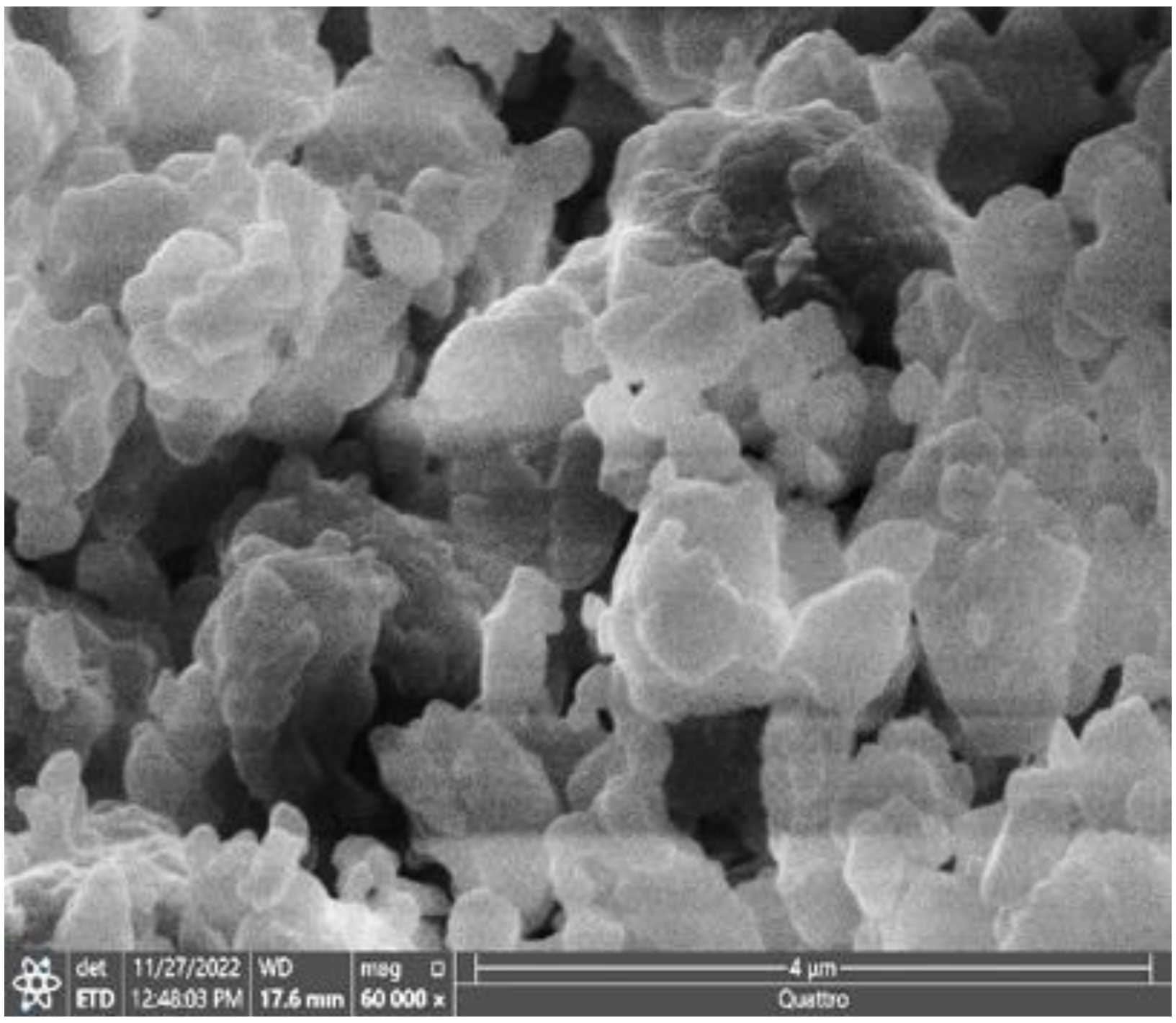

Figure S1. Scanning electron micrograph of nano-melatonin. The image shows irregularly shaped nano-melatonin particles forming aggregated clusters. The micrograph was obtained at ×60,000 magnification with a 4 μm scale bar.

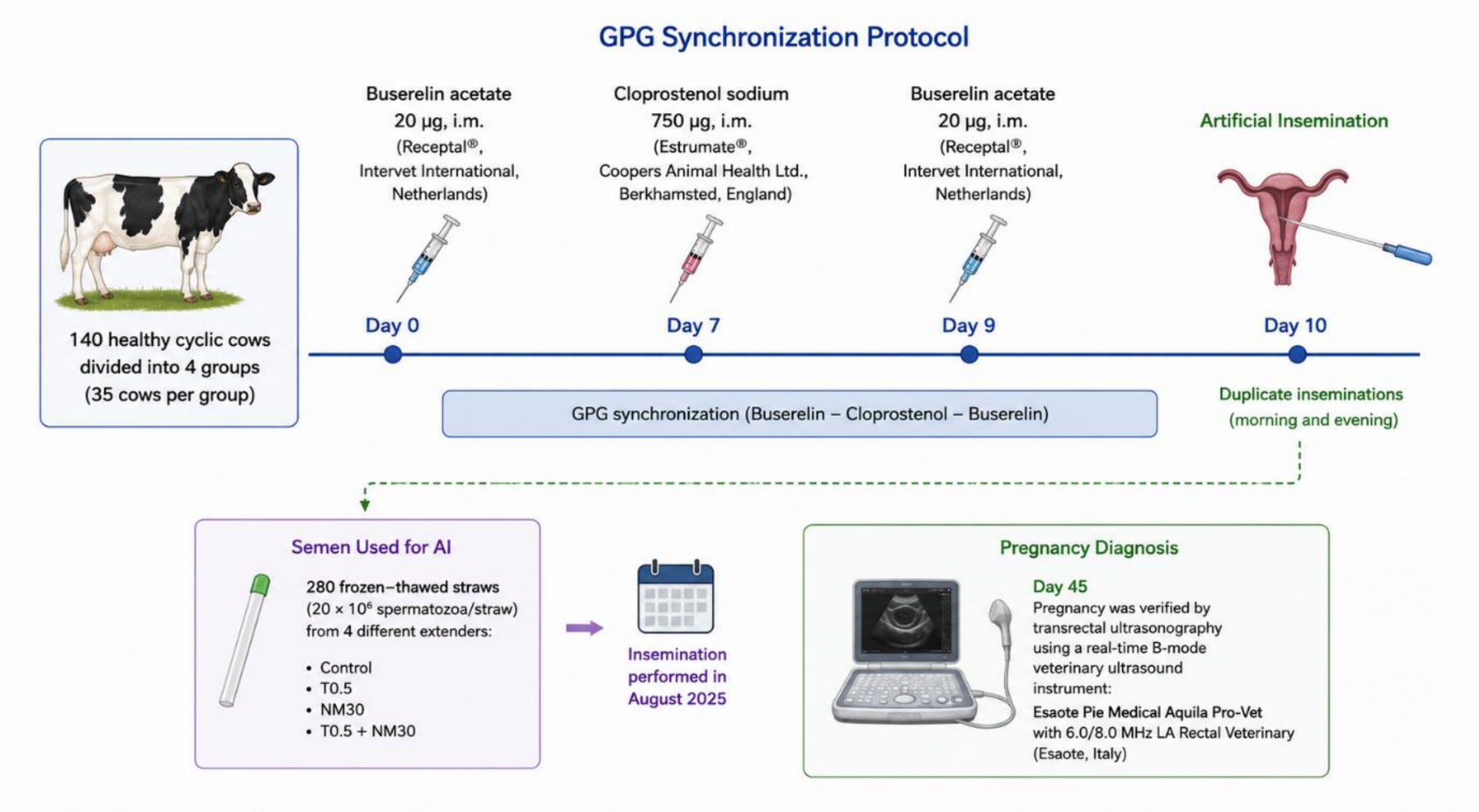

Figure S2. GPG synchronization protocol, artificial insemination, and pregnancy diagnosis in cyclic cows. A total of 140 healthy cyclic cows were allocated to four groups (n = 35 per group). Estrus synchronization comprised intramuscular administration of buserelin acetate (20 µg) on day 0, cloprostenol sodium (750 µg) on day 7, and a second dose of buserelin acetate (20 µg) on day 9. On day 10, cows underwent duplicate artificial inseminations in the morning and evening using frozen–thawed semen containing 20×10^6 spermatozoa per straw. Semen was prepared with one of four extenders: control, thyroxine (T0.5), nano-melatonin (NM30), or their combination (T0.5 + NM30). Pregnancy was diagnosed on day 45 by transrectal B-mode ultrasonography. GPG, gonadotropin-releasing hormone–prostaglandin $F_2\alpha$–gonadotropin-releasing hormone; AI, artificial insemination.
